# Supplementary material for: Sir2 and Fun30 regulate ribosomal DNA replication timing via MCM helicase positioning and nucleosome occupancy
Source: bioRxiv. 2024 Oct 28:2024.03.21.586113. Originally published 2024 Mar 26. Preprint. [Version 3] doi: 10.1101/2024.03.21.586113 (PMC10996493; doi:10.1101/2024.03.21.586113)
Supplement: Supplement 1 [file NIHPP2024.03.21.586113v3-supplement-1.pdf]

bioRxiv preprint doi: https://doi.org/10.1101/2024.03.21.586113; this version posted October 28, 2024. The copyright holder for this preprint (which was not certified by peer review) is the author/funder, who has granted bioRxiv a license to display the preprint in perpetuity. It is made available under aCC-BY 4.0 International license.

Figure 1-figure supplement 1

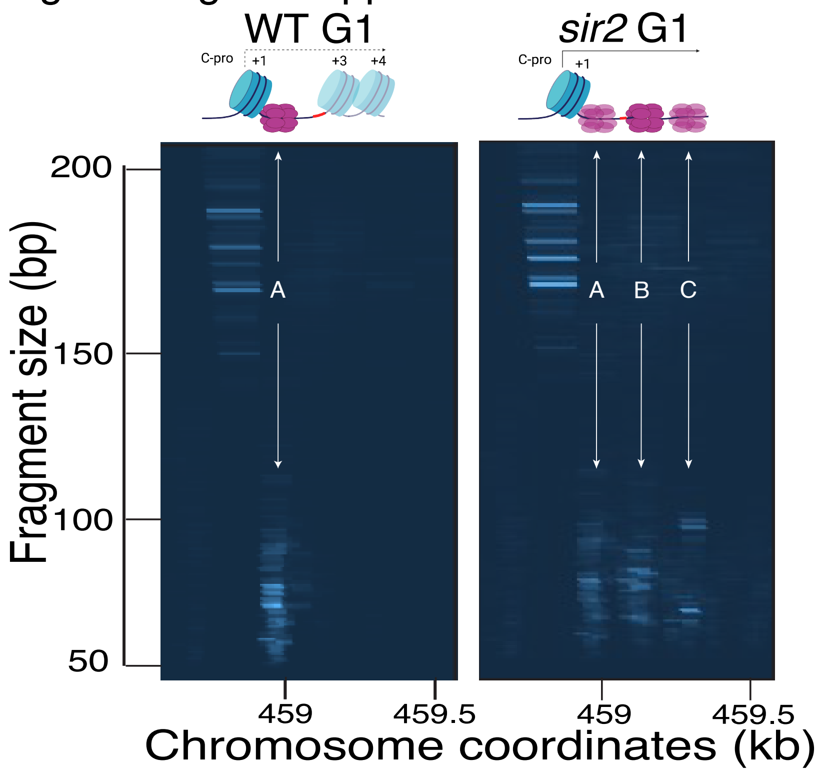

Figure 1-figure supplement 1. Computer-generated visualization of non-displaced and displaced MCM complexes, as determined by MCM2-ChEC. Sequencing reads from G1 cultures processed for MCM2-ChEC for wild type (16747) (left) and *sir2* (16769) (right) were plotted according to genomic location (x axis) and library insert size (y axis), with read depths indicated by color intensity. Note that these images are not agarose gels. The signal generated from inserts in the 50-100 base pair range (y axis) reflects the MCM complexes, whereas the signal from inserts in the 150-200 base pair range reflects the +1 nucleosome (see main text). De-repression of C-pro transcription in *sir2* causes RNA polII to push the MCM helicase complex from its normal location (arrow labeled "1") to the right, with the most prominent signals arising at the sites indicated by arrows labeled "2" and "3". Note that the presence of multiple MCM footprints in these composite images does not indicate that the presence of multiple MCM complexes in any individual repeat.

bioRxiv preprint doi: https://doi.org/10.1101/2024.03.21.586113; this version posted October 28, 2024. The copyright holder for this preprint (which was not certified by peer review) is the author/funder, who has granted bioRxiv a license to display the preprint in perpetuity. It is made available under aCC-BY 4.0 International license.

Figure 2-figure supplement 1

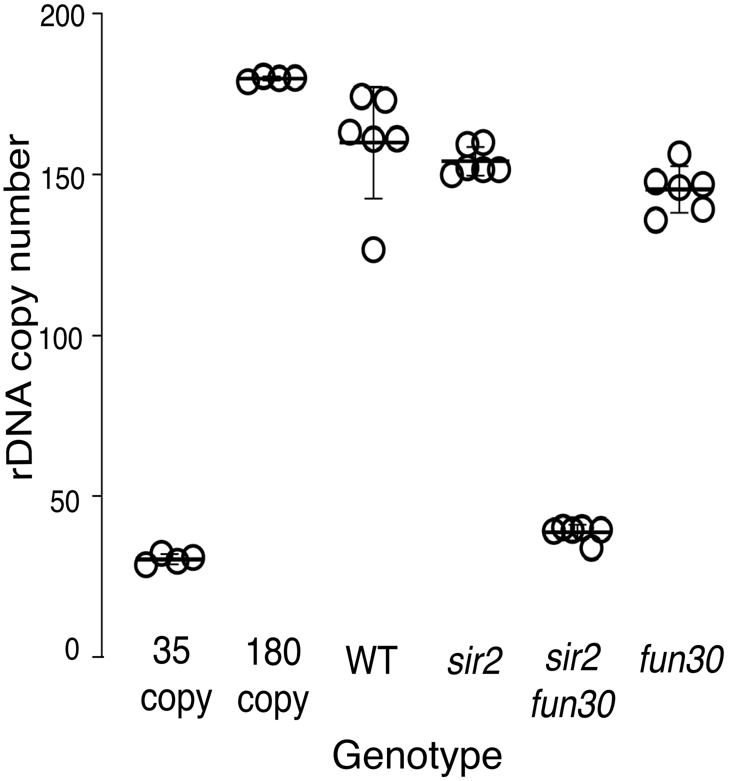

Figure 2-figure supplement 1. rDNA size as determined by qPCR. rDNA size for multiple isolates from each genotype were determined by qPCR, as described (Materials and Methods). Control strains with 35 (EK342) and 180 (EK68) copies of the rDNA were generated in *fob1* backgrounds to ensure copy number stability and were used as standards. Copy numbers were 160±7, 156±7, 38±3 and 147±7 for WT (14141), *sir2* (16668), *sir2 fun30* (17263) and *fun30* (17248), respectively ($p<0.001$ by t-test for *sir2* vs *sir2fun30*).

bioRxiv preprint doi: https://doi.org/10.1101/2024.03.21.586113; this version posted October 28, 2024. The copyright holder for this preprint (which was not certified by peer review) is the author/funder, who has granted bioRxiv a license to display the preprint in perpetuity. It is made available under aCC-BY 4.0 International license.

Figure 2-figure supplement 2

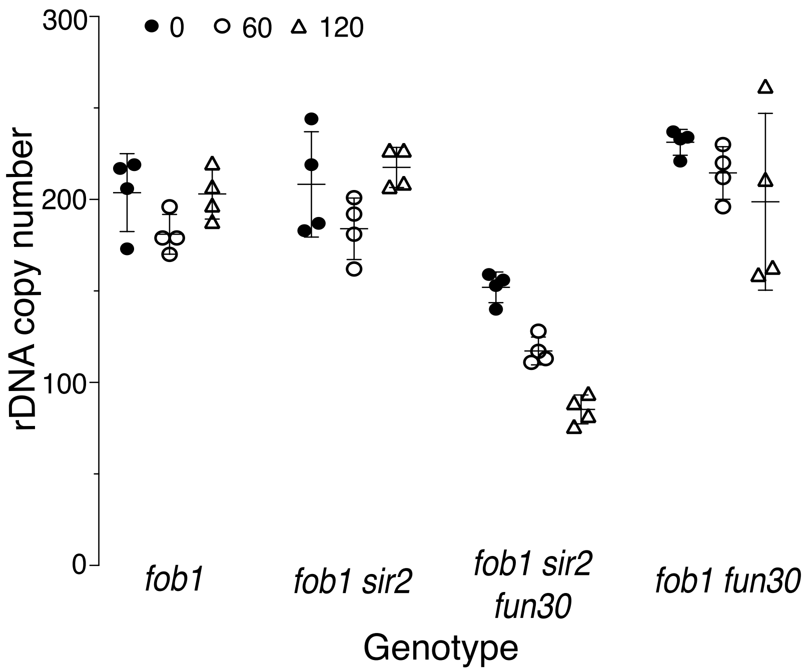

Figure 2-figure supplement 2. Changes in rDNA size of *fob1* strains with continuous passaging. Changes in rDNA size with passaging for 0 (closed circles), 60 (open circles) and 120 (open triangles) generations were determined by qPCR, as described (Materials and Methods) for the following genotypes (strain numbers in parentheses): *fob1 fun30* (17476), *fob1 sir2 fun30* (17548), *fob1* (17549), and *fob1 sir2* (17562). Control strains with 35 (EK342) and 180 (EK68) copies of the rDNA repeat were used as standards. The copy numbers (mean±SD) at 0, 60 and 120 divisions for different genotypes were as follows: 204±21, 191±22 and 203±14 for *fob1* (p NS by two-tailed t-test for any pairwise comparison); 208±29, 190±27 and 218±11 for *sir2 fob1* (p NS by t-test for any pairwise comparison); 152±8, 107±22 and 85±8 for *sir2 fun30 fob1* (p <0.001 by t-test for any pairwise comparison); and 231±7, 225±28 and 199±48 for *fun30 fob1* (p NS by t-test for any pairwise comparison).

bioRxiv preprint doi: https://doi.org/10.1101/2024.03.21.586113; this version posted October 28, 2024. The copyright holder for this preprint (which was not certified by peer review) is the author/funder, who has granted bioRxiv a license to display the preprint in perpetuity. It is made available under aCC-BY 4.0 International license.

Figure 2-figure supplement 3

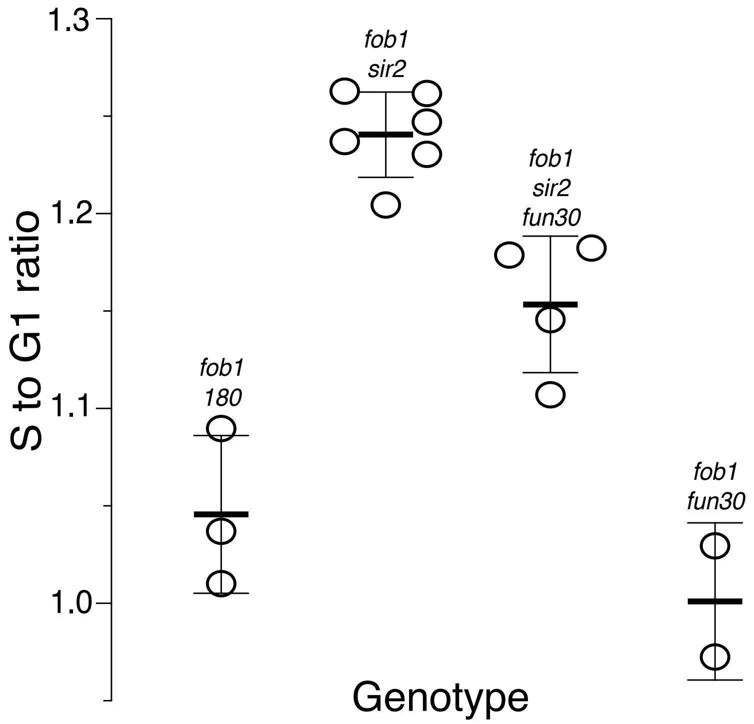

Figure 2-figure supplement 3. rDNA replication timing in *fob1* strains. Relative timing (Trel) of replication of the rDNA was determined as described in the main text. A value of 1 indicates that replication occurred at the genome-wide average; higher or lower values reflect earlier or later replication, respectively. Strains numbers used were as follows: *fob1* (16630, 17549, and 17550), *fob1 sir2* (17561), *fob1 sir2 fun30* (17548), and *fob1 fun30* (17477 and 17542). Trel values (mean±SD) were 1.05±0.04 for *fob1*, 1.24±0.02 for *fob1 sir2*, 1.15±0.04 for *fob1 sir2 fun30* and 1.00±0.04 for *fob1 fun30;* $p<0.01$ by t-test for *fob1 sir2* vs *fob1 sir2 fun30* and for *fob1* vs *fob1 sir2.*

bioRxiv preprint doi: https://doi.org/10.1101/2024.03.21.586113; this version posted October 28, 2024. The copyright holder for this preprint (which was not certified by peer review) is the author/funder, who has granted bioRxiv a license to display the preprint in perpetuity. It is made available under aCC-BY 4.0 International license.

Figure 3-figure supplement 1

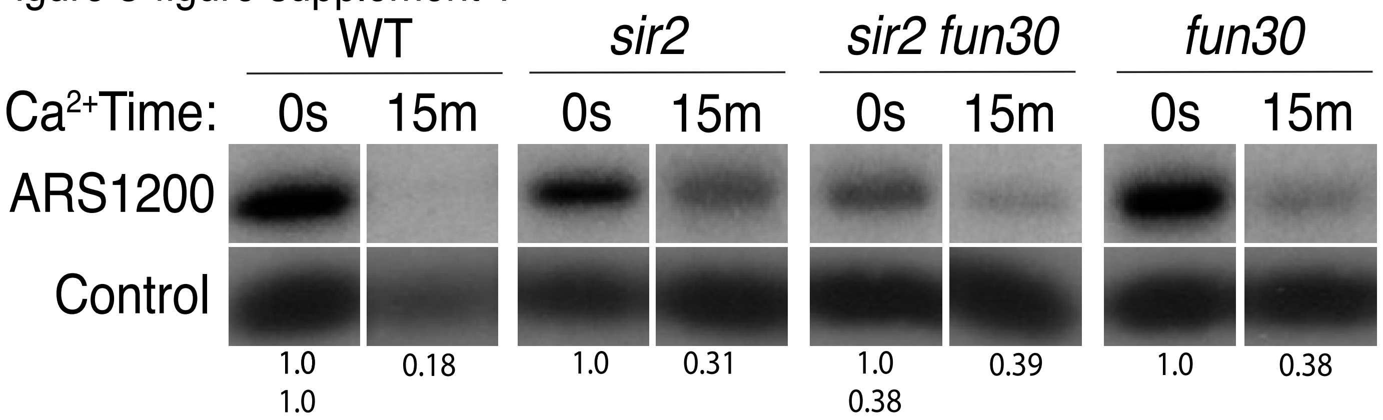

Figure 3- figure supplement 1. Licensing at the rARS using Southern blot (Replica of Figure 3). Activation of MCM-MNase in G1-arrested cells. PIK1 serves as a loading control. Normalized ARS1200 band intensity at 15 minutes is expressed relative to time 0. Quantitation of the uncut band was used to infer relative rDNA array size in *sir2 fun30* mutant at 0.38 relative to WT.

bioRxiv preprint doi: https://doi.org/10.1101/2024.03.21.586113; this version posted October 28, 2024. The copyright holder for this preprint (which was not certified by peer review) is the author/funder, who has granted bioRxiv a license to display the preprint in perpetuity. It is made available under aCC-BY 4.0 International license.

Figure 5- figure supplement 1

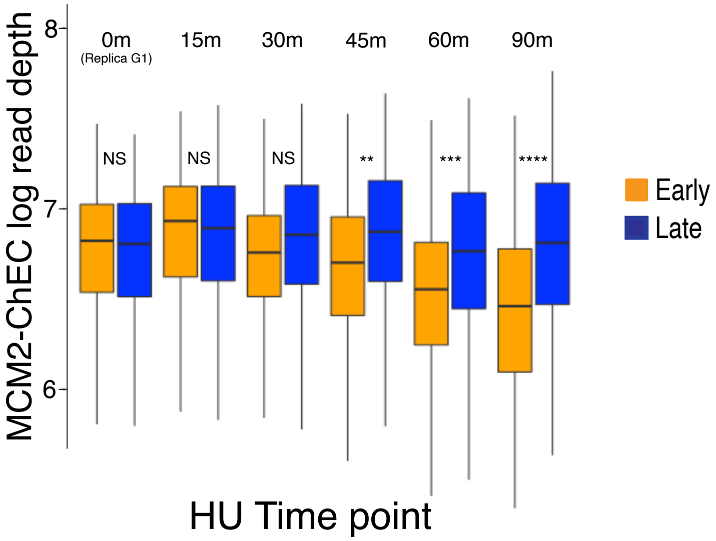

Figure 5-figure supplement 1. Quantification of MCM2-ChEC signal at 111 early (orange) and 101 late (blue) origins as cells progress through S phase (quantitation of Figure 5B). Cells were arrested in G1, released into medium containing 200 mM HU, and analyzed by MCM2-ChEC-seq at different time points. Total genome-wide read counts for each sample were normalized to the genome-wide read counts for the sample with the highest count. Strain is the same one used in Figure 5B. At each time point, we used Student's t test to determine MCM2-ChEC signal was different between early and late origins. Significance of two tail t-tests are abbreviated as "NS" (not significant), **($p \leq 0.01$), ***($p \leq 0.001$), and **** ($p \leq 0.0001$).

bioRxiv preprint doi: https://doi.org/10.1101/2024.03.21.586113; this version posted October 28, 2024. The copyright holder for this preprint (which was not certified by peer review) is the author/funder, who has granted bioRxiv a license to display the preprint in perpetuity. It is made available under aCC-BY 4.0 International license.

Figure 5- figure supplement 2

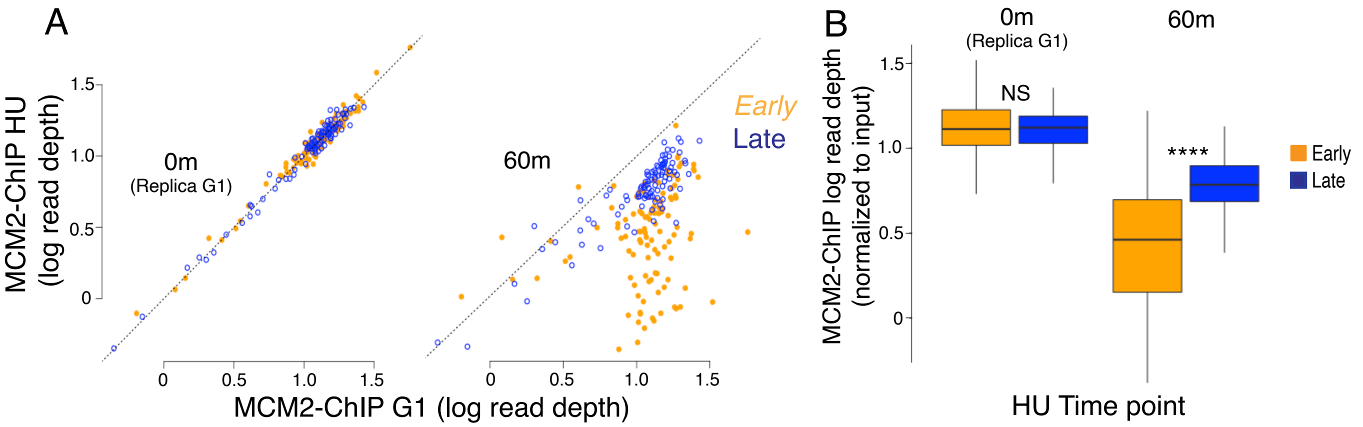

Figure 5-figure supplement 2. Quantitation of early and late origins using MCM2-ChIP. Chromatin-IP of FLAG-tagged MCM2 was used to measure levels of 111 early and 101 late origins in G1 and 60 minutes after release from G1 into medium containing 200 mM HU. Analysis was performed as done with MCM2-ChEC in Figure 5B and Figure 5-figure supplement 1. **A.** The MCM2-ChIP signal over a 200 base pair window at 111 early (orange) and 101 late (blue) origins in WT (17558) G1-arrested cells is compared to the signal in cells released into hydroxyurea for 60 minutes. The signal at early origins diminishes more than the signal at late origins. **B.** Box plots for MCM2-ChIP signal at early and late origins. P-values by two tail t-test for the signal abundance at early vs late origins: **** ($p \leq 0.0001$).

bioRxiv preprint doi: https://doi.org/10.1101/2024.03.21.586113; this version posted October 28, 2024. The copyright holder for this preprint (which was not certified by peer review) is the author/funder, who has granted bioRxiv a license to display the preprint in perpetuity. It is made available under aCC-BY 4.0 International license.

Figure 5- figure supplement 3

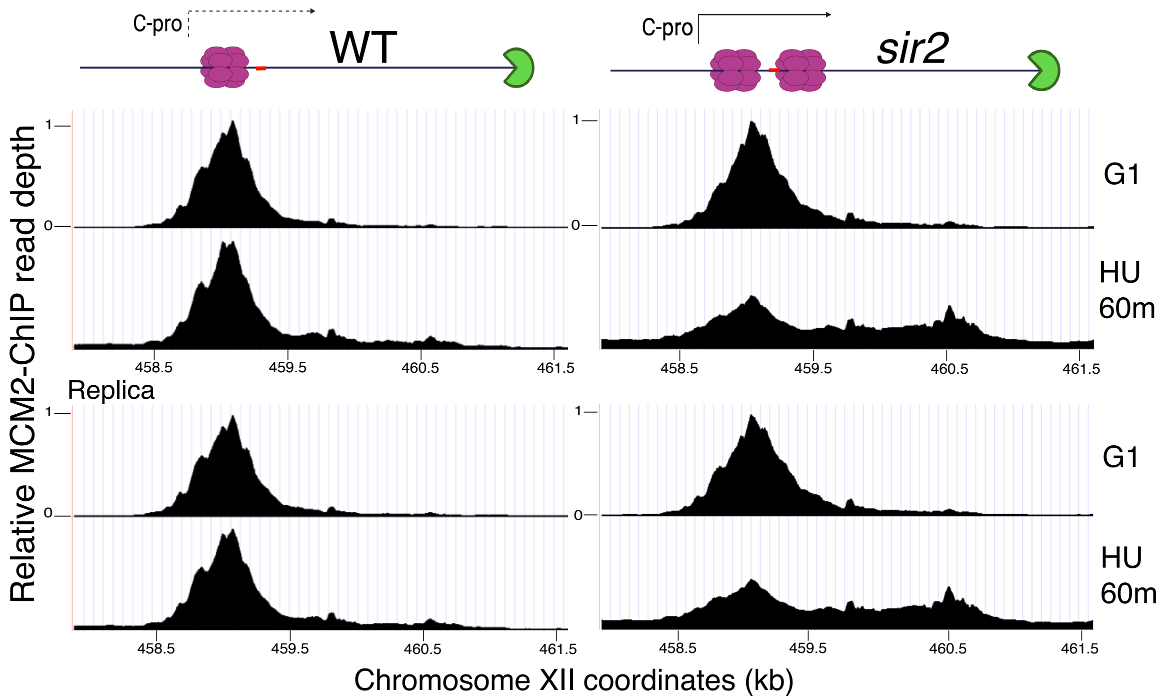

Figure 5- figure supplement 3. Change in localization of MCM2-ChIP signal at the rDNA with progression from G1 into S phase. Wild-type (left; strain 17558) and *sir2* (right; strain 17559) cells with FLAG-tagged MCM2 were arrested in G1 prior to release into media containing 200 mM HU and analyzed by ChIP. Relative MCM2-CHIP read depth is plotted according to chrXII coordinates. Diagrams at top show MCM double-hexamers in purple, the RFB in green, and the location of the C-pro transcript, either transcribed (solid line) or repressed (dotted line).

bioRxiv preprint doi: https://doi.org/10.1101/2024.03.21.586113; this version posted October 28, 2024. The copyright holder for this preprint (which was not certified by peer review) is the author/funder, who has granted bioRxiv a license to display the preprint in perpetuity. It is made available under aCC-BY 4.0 International license.

Figure 5-figure supplement 4

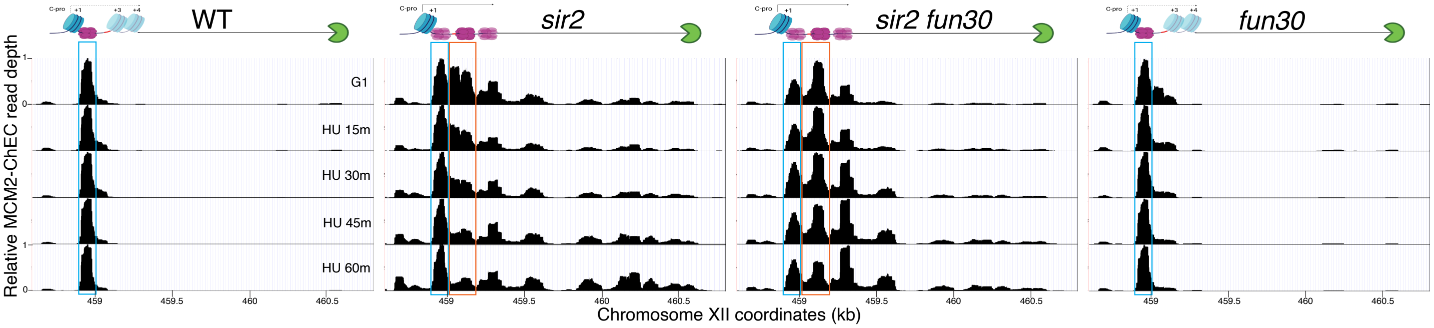

Figure 5-figure supplement 4. Deletion of *FUN30* suppresses activation of the displaced MCM complex at the rDNA (replica of results in Figure 5C). Each panel shows MCM2-ChEC data for cultures that were arrested in G1 and then released into medium containing 200 mM HU. Non-displaced and displaced MCM complexes are indicated by blue and orange rectangles, respectively. MCM2-ChEC data were quantified for the 51-100 base pair size range. Strains are the same as those used in Figure 5C. Diagrams at top show MCM double-hexamers in purple, the RFB in green, nucleosomes in blue, and the location of the C-pro transcript, either transcribed (solid line) or repressed (dotted line).

bioRxiv preprint doi: https://doi.org/10.1101/2024.03.21.586113; this version posted October 28, 2024. The copyright holder for this preprint (which was not certified by peer review) is the author/funder, who has granted bioRxiv a license to display the preprint in perpetuity. It is made available under aCC-BY 4.0 International license.

Figure 5-figure supplement 5

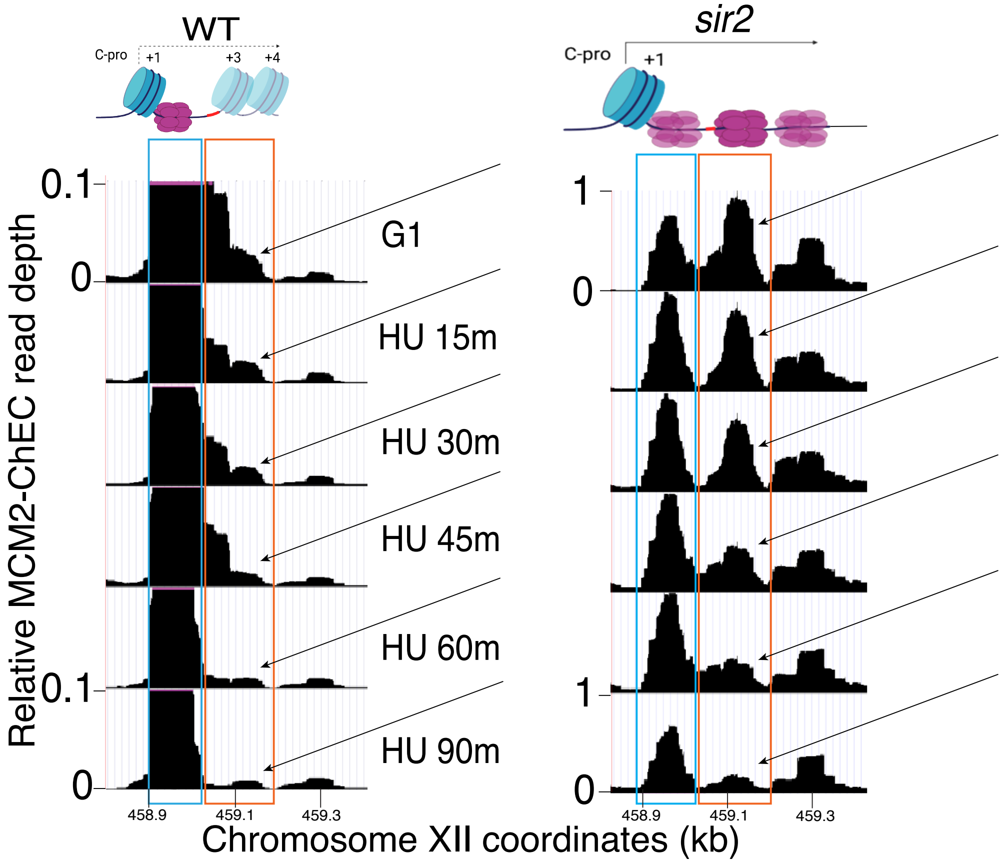

Figure 5-figure supplement 5. Displaced MCM complex is activated early in wild type. Enlarged region from figure 5C shows that, although only a small proportion of MCM complexes are displaced in WT (16747), this displaced population, indicated by arrows, is activated early, as is the case with in *sir2* (16769). Cartoons at top show nucleosomes in blue, MCM double-hexamers in purple, C-pro transcription, or lack thereof, as solid or dotted lines, respectively.

bioRxiv preprint doi: https://doi.org/10.1101/2024.03.21.586113; this version posted October 28, 2024. The copyright holder for this preprint (which was not certified by peer review) is the author/funder, who has granted bioRxiv a license to display the preprint in perpetuity. It is made available under aCC-BY 4.0 International license.

Figure 5-figure supplement 6

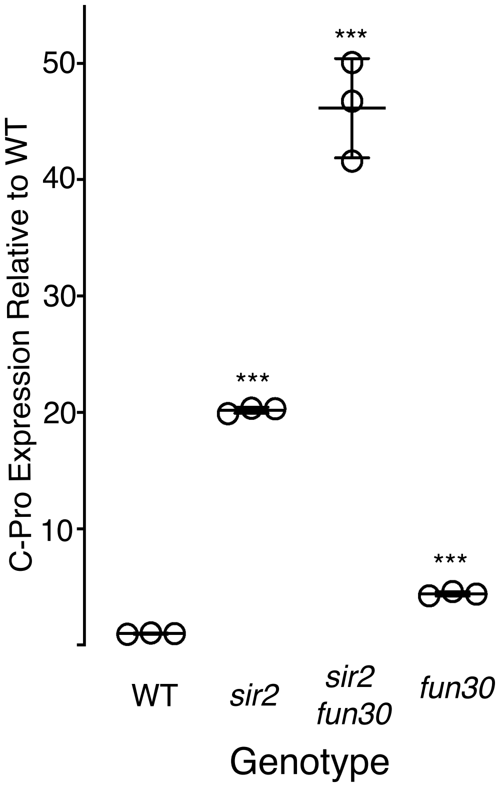

Figure 5-figure supplement 6. C-pro transcript levels. C-pro transcript levels in G1-arrested cells in *sir2* (16316), *sir2 fun30* (16727), and *fun30* (16711) were measured in triplicate using qPCR and expressed relative to WT (14141). All values are normalized to rDNA array size. *** denotes p<0.001 for comparison with WT using t-test.

bioRxiv preprint doi: https://doi.org/10.1101/2024.03.21.586113; this version posted October 28, 2024. The copyright holder for this preprint (which was not certified by peer review) is the author/funder, who has granted bioRxiv a license to display the preprint in perpetuity. It is made available under aCC-BY 4.0 International license.

Figure 6-figure supplement 1

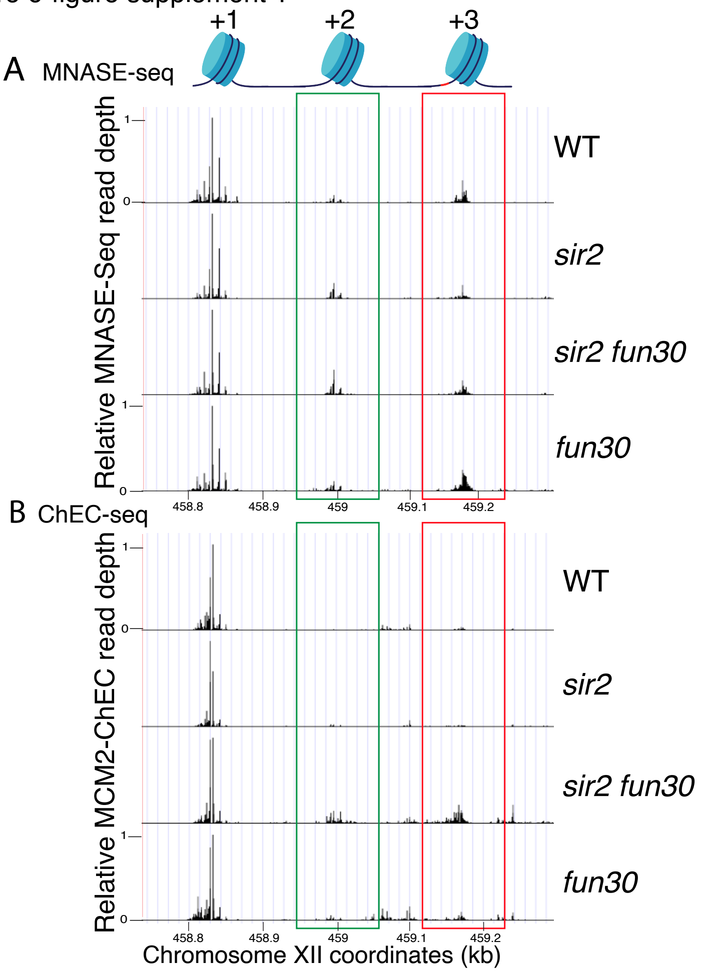

Figure 6-figure supplement 1. Nucleosome occupancy assessed by MNase-seq and MCM2-ChEC (Replica of Figure 6). A. MNase-seq analysis of nucleosome occupancy at rDNA origins Deletion of *FUN30* increased occupancy at +2 (green) and +3 (red) positions in both *sir2* and *SIR2* backgrounds. Strains are the same as those used in Figure 6. **B.** MCM2-ChEC analysis confirmed that *FUN30* deletion increases +2 and +3 nucleosome occupancy in a *sir2* background.
