# Supplementary material for: Sir2 and Fun30 regulate ribosomal DNA replication timing via Mcm helicase positioning and nucleosome occupancy
Source: bioRxiv. 2024 Mar 26:2024.03.21.586113. Preprint. [Version 1] doi: 10.1101/2024.03.21.586113 (PMC10996493; doi:10.1101/2024.03.21.586113)
Supplement: Supplementary file 1 [file NIHPP2024.03.21.586113V1-supplement-1.pdf]

bioRxiv preprint doi: https://doi.org/10.1101/2024.03.21.586113; this version posted March 26, 2024. The copyright holder for this preprint (which was not certified by peer review) is the author/funder, who has granted bioRxiv a license to display the preprint in perpetuity. It is made available under aCC-BY 4.0 International license.

Table S1: Yeast strains

Strain	Genotype
W1588-4c*	*MATa, ade2-1, Can1-100, his3-11,15, leu2-3,112, trp1-1, ura3-1*, *RAD5*+, W303 background
16535	*MATa, his3, leu2, met15, ura3, hmlα*::HYG
16668	*MATa, his3, leu2, met15, ura3, hmlα*::HYG, *sir2*::KanMX
16673	*MATa, his3, leu2, met15, ura3, hmlα*::HYG, *isw2*::NAT, *sir2*::LEU
16723	*MATa, his3, leu2, met15, ura3, irc5*::KanMX, *sir2*::LEU
16724	*MATa, his3, leu2, met15, ura3, isw1*::KanMX, *sir2*::LEU
16725	*MATa, his3, leu2, met15, ura3, chd1*::KanMX, *sir2*::LEU
16728	*MATa, his3, leu2, met15, ura3, swr1*::KanMX, *sir2*::HYG
16729	*MATa, his3, leu2, met15, ura3, nhp10*::KanMX, *sir2*::LEU
16747	*MATa, his3, leu2, met15, ura3, hmlα*::HYG, with *MCM2* MNase 3xFlag Tag with KanMx (161 rDNA copies)
16769	*MATa, his3, leu2, met15, ura3, hmlα*::HYG, *sir2*::LEU, with *MCM2* MNase 3xFlag Tag with KanMx (152 rDNA copies)
16909	*MATa, his3, leu2, met15, ura3, fun30*::KanMX, *sir2*::LEU, *hmlα*::HYG
17059	*MATa, his3, leu2, met15, ura3, htl1*::NAT, *sir2*::LEU
17113	*MATa, his3, leu2, met15, ura3, hmlα*::HYG, *fun30S20AS28A*, *sir2*::LEU (164 rDNA copies)
17114	*MATa, his3, leu2, met15, ura3, hmlα*::HYG, *fun30S20AS28A*, *sir2*::LEU (166 rDNA copies)
17248	*MATa, his3, leu2, met15, ura3, hmlα*:: HYG, *fun30*::URA
17256	*MATa, his3, leu2, met15, ura3, hmlα*::HYG, *fun30*::NAT, with *MCM2* MNase 3xFlag Tag with KanMx (146 rDNA copies)
17257	*MATa, his3, leu2, met15, ura3, hmlα*::HYG, *sir2*::LEU, *fun30*::NAT, with *MCM2* MNase 3xFlag Tag with KanMx (39 rDNA copies)
17265	*MATa, ade2-1, Can1-100, his3-11,15, leu2-3,112, trp1-1, ura3-1*, *RAD5*+, with p306-BrdU-Inc, p403-BrdU-Inc, p405-BrdU-Inc, p404-BrdU-Inc, *hmlα*::HYG, W303 background
17271	*MATa, ade2-1, Can1-100, his3-11,15, leu2-3,112, trp1-1, ura3-1*, *RAD5*+, with p306-BrdU-Inc, p403-BrdU-Inc, p405-BrdU-Inc, p404-BrdU-Inc, *hmlα*::HYG, *sir2*::KanMX, W303 background
17279	*MATa, ade2-1, Can1-100, his3-11,15, leu2-3,112, trp1-1, ura3-1*, *RAD5*+, with p306-BrdU-Inc, p403-BrdU-Inc, p405-BrdU-Inc, p404-BrdU-Inc, *hmlα*::HYG, *sir2*::KanMX, *fun30*::NAT, W303 background
17281	*MATa, ade2-1, Can1-100, his3-11,15, leu2-3,112, trp1-1, ura3-1*, *RAD5*+, with p306-BrdU-Inc, p403-BrdU-Inc, p405-BrdU-Inc, p404-BrdU-Inc, *hmlα*::HYG, *fun30*::NAT, W303 background
17345	*MATa, his3, leu2, met15, ura3, hmlα*::HYG, *fun30K603R, sir2*::LEU
17346	*MATa, his3, leu2, met15, ura3, hmlα*::HYG, *fun30K603R, sir2*::LEU
EK342**	*MATa, his3, leu2, met15, ura3, fob1*::NAT; BY rDNA (35 rDNA copies)
EK68**	*MATa*, Prototroph, *fob1*::NAT; BY rDNA (180 rDNA copies)

*Strain from Toshi Tsukiyama (Rodriguez et al. 2017)

** Strain from Bonny Brewer and M.K. Raghuraman (Kwan et al. 2023)

bioRxiv preprint doi: https://doi.org/10.1101/2024.03.21.586113; this version posted March 26, 2024. The copyright holder for this preprint (which was not certified by peer review) is the author/funder, who has granted bioRxiv a license to display the preprint in perpetuity. It is made available under aCC-BY 4.0 International license.

Table S2: Primers used

Primers	Sequence (5' to 3')	Comments
5P_c_pro_qPCR	CCATCTATGTCTTCCACACC	5' primer for C-pro qRT-PCR
3P_c_pro_qPCR	GTCATTTACAAGAGGTAGGTCG	3' primer for C-pro qRT-PCR
PDA1_1_F	GCTGCTTCATTCAAACGCCA	5' primer for *PDA1* control for C-pro qRT-PCR
PDA1_1_R	CTGGTGGGAGTGCGAAGAAC	3' primer for *PDA1* control for C-pro qRT-PCR
Tor2_5p_qPCR	GCACAAAGACCACAAAGTCG	5' primer for *TOR2* control for rDNA size qPCR
Tor2_3p_qPCR	GGGGATAGAGAACTAACAAAAGCA	3' primer for *TOR2* control for rDNA size qPCR
ARS1200_qPCR_Forward	GAAATACGCTTCAGAGACCC	5' primer for ARS1200 for rDNA size qPCR
ARS1200_qPCR_Reverse	CGGAAATGGCTGATTTTGATTG	3' primer for ARS1200 for rDNA size qPCR
rARS_southern_5prime1	ACTTCAATTTAAGCTATTCTCC	5' primer for ARS1200 probe for Southern Blot
rARS_3Primer_southern_1	TGCAAGTAGCATATATTTCTTG	3' primer for ARS1200 probe for Southern Blot
Pik1_F	GATTCAGCGTCCACAGCTC	5' primer for *PIK1* control probe for Southern Blot
Pik1_B	GGTAACCAAATTTATCCTGCACC	3' primer for *PIK1* control probe for Southern Blot
